# Extracellular vesicle-based therapeutics: natural versus engineered targeting and trafficking

**DOI:** 10.1038/s12276-019-0223-5

**Published:** 2019-03-15

**Authors:** Daniel E. Murphy, Olivier G. de Jong, Maarten Brouwer, Matthew J. Wood, Grégory Lavieu, Raymond M. Schiffelers, Pieter Vader

**Affiliations:** 1 Laboratory of Clinical Chemistry and Haematology, UMC Utrecht, Utrecht, The Netherlands; 2 Department of Physiology, Anatomy and Genetics, University of Oxford, Oxford, UK; 3 Institut Curie, PSL Research University, INSERM U932 Paris, France; 4 Department of Experimental Cardiology, UMC Utrecht, Utrecht, The Netherlands

**Keywords:** Cell signalling

## Abstract

Extracellular vesicles (EVs) are increasingly being recognized as mediators of intercellular signaling via the delivery of effector molecules. Interestingly, certain types of EVs are also capable of inducing therapeutic responses. For these reasons, the therapeutic potential of EVs is a topic of intense research, both in the context of drug delivery and regenerative medicine. However, to fully utilize EVs for therapeutic purposes, an improved understanding of the mechanisms by which they function would be highly advantageous. Here, the current state of knowledge regarding the cellular uptake and trafficking of EVs is reviewed, along with a consideration of how these pathways potentially influence the functions of therapeutic EVs. Furthermore, the natural cell-targeting abilities, biodistribution profiles, and pharmacokinetics of exogenously administered EVs, along with the components responsible for these features are discussed. An overview of the potential clinical applications and preclinical examples of their successful use is also provided. Finally, examples of EV modifications that have successfully been employed to improve their therapeutic characteristics receive a particular focus. We suggest that, in addition to investigation of EV cell targeting and routes of uptake, future research into the routes of intracellular trafficking in recipient cells is required to optimally utilize EVs for therapeutic purposes.

## Therapeutics: Helping vesicles to deliver drugs inside cells

An increased understanding of how extracellular vesicles (EVs) enter cells and deliver molecules will enable promising new therapies, according to researchers in the Netherlands, UK and France. EVs are liquid-filled sacs secreted by cells that transport proteins, lipids and RNA between cells, and therefore have potential for delivering drugs. Pieter Vader at UMC Utrecht and co-workers review recent research into EVs, focusing on how EVs are distributed around the body, and how they target and enter cells. However, there is little known about EV biology once they are inside cells, and it is likely that many EVs simply degrade without delivering their cargo. Further research in this area could help identify features that improve cargo escape from EVs, thus ensuring that future therapies can be effective.

## Introduction

A major limiting factor in the development and application of biologicals is an appropriate method for the delivery of these molecules to their sites of action. For example, RNA therapeutics possess great potential due to their ability to alter gene expression; however, the large polar RNA molecule is unable to cross the cell membrane and is subject to rapid digestion by extracellular RNases. Current conventional strategies designed to overcome these barriers involve enveloping therapeutic RNA within synthetic nanoparticles^1^ or conjugating RNA to specific ligands designed to promote uptake^2^. More recently, the FDA has approved lipid nanoparticles (LNPs) developed by Alnylam for the delivery of siRNAs to treat hereditary transthyretin amyloidosis, representing the first-ever approved drug based on RNA interference. LNPs protect their delicate cargo from degradation and facilitate entry into cells. Despite overcoming these obstacles, LNPs can display (dose-limiting) toxicity and LNP-mediated RNA delivery is largely limited to the liver^3^. Thus, ineffective delivery to other tissues continues to delay the development of effective therapeutic strategies.

A solution to this drug delivery problem is urgently needed, and for this reason, the therapeutic potential of extracellular vesicles (EVs) has become a topic of intense research. These lipid-bound particles with a diameter of 30–1000 nm facilitate intercellular communication following their release from donor cells and subsequent internalization into surrounding or distant recipient cells. This process results in the transfer of their protein, lipid, and RNA cargo, thereby eliciting a response in recipient cells^4^. Due to this ability to function as an endogenous intercellular cargo transfer system, EVs have been studied for use as potential vehicles for drug delivery. In addition to their ability to deliver exogenous therapeutic cargo, certain EVs also possess inherent therapeutic characteristics, most notably in the context of regenerative medicine^5^.

In order to understand how EVs can be optimally utilized for therapeutic purposes, it is important to understand the processes by which they are formed and how they function in health and disease. EVs are classed into two major subtypes based on their biogenesis—exosomes and microvesicles. Exosomes range from 30–100 nm in diameter and are derived from endosomal compartments^6^. In contrast, microvesicles are formed through a process of budding and pinching off from the cell membrane^7^. They are highly heterogeneous in size and vary from 50–1000 nm in diameter.

Exosomes are formed within multivesicular endosomes (MVEs) through a process of inward budding, resulting in the formation of intraluminal vesicles (ILVs). These ILVs are released into the extracellular environment—at which point they are termed exosomes—upon the maturation of the MVE and subsequent fusion with the cell surface. This process depends on numerous pathways, many of which have not yet been fully elucidated; however, proteins with important roles in this process are the endosomal sorting complexes required for transport (ESCRT) protein family^8^. Various members of the ESCRT family are responsible for the organization of proteins on the MVE membrane and the eventual budding off into the MVE lumen, which is assisted by ESCRT-III^9^. ILVs are also generated through a process that does not require the ESCRT complex. An important factor required for ESCRT-independent ILV formation is the tetraspanin CD63^10^. CD63 is a typical EV protein marker, and its importance in EV biogenesis is highlighted by the observation that its knockout results in decreased EV release from human embryonic kidney 293 (HEK293) cells^11^. Another important mediator of ESCRT-independent ILV formation is neutral sphingomyelinase 2. This enzyme is responsible for the synthesis of the lipid ceramide, which forms microdomains on the MVE membrane, a process that is crucial for ILV generation^12^. The formation of these ceramide-rich lipid microdomains is followed by an inward invagination that may be promoted by the cone-like shape of the ceramide molecule^13^. Other proteins have recently been shown to be involved in exosome formation. Hsp90, a chaperone protein, appears to exhibit specific MVE-related activity that triggers the deformation of the MVE, fusion of the MVE with the plasma membrane and subsequent release of exosomes^14^. Further studies are needed to elucidate whether microvesicle formation is a completely Hsp90-independent process.

Similarly to exosomes, the secretion of microvesicles is also partially ESCRT dependent and requires the formation of lipid microdomains at the plasma membrane. The ESCRT-1 component tumor susceptibility gene 101 has been reported to interact with arrestin domain-containing protein 1 at the cell surface, which mediates the budding off of microvesicles^15^. In addition, the formation of ceramide-rich lipid microdomains is also involved in microvesicle formation^16^. In contrast to exosome formation, in which ESCRT-III is responsible for the scission of ILVs into the MVE lumen, a reorganization of the actin-myosin cytoskeletal network mediated by ADP-ribosylation factor 6 is the mechanism by which budding microvesicles are released from the plasma membrane^17^.

EVs are loaded with a diverse range of proteins, some of which are common to most EV subsets released from most cell types, such as the membrane-bound tetraspanins CD9, CD81, and CD63^18^. Others are detected in EVs derived from only a specific subset of cell types, such as the truncated form of epidermal growth factor receptor known as EGFRvIII, which has been identified on the surface of glioma-derived EVs^19^.

In addition to their protein contents, EVs are also loaded with nucleic acids. For example, there are examples of mRNA transcripts loaded within EVs which can be functionally transferred from producer to recipient cells^20,21^. Noncoding RNAs (ncRNAs) such as microRNAs (miRNAs) are also loaded in EVs, some of which are enriched in comparison to their cells of origin^22^. The incorporation of ncRNAs is also influenced by the status of the producing cell. For example, immune activation has been shown to influence the ncRNA transcriptome of dendritic cell-derived EVs^23^. These observations suggest that the ncRNA content of EVs plays a physiological role. Furthermore, various types of DNA, such as mitochondrial and genomic DNA have also be found within EVs^24,25^.

The lipid composition of EVs is markedly different from the composition of the cell membrane. EVs can be enriched in phosphatidylserine (PS)^26^, lipids that positively regulate the curvature of the outer membrane positive such as lysophosphatidylcholine and lipids that negatively regulate the curvature of the inner membrane such as cardiolipin^27^. The lipids contained in EVs may exert a signaling function. For example, eicosanoids present in EVs, such as prostaglandins and leukotrienes, have been implicated in signaling processes^28^.

In most cases, the precise mechanisms by which EVs exert their functions remain to be elucidated. However, some EVs transfer active cargo that has been specifically shown to induce a response in recipient cells. Functional transfer of chemokine receptor 5 (CCR5) by EVs from CCR5+cells to CCR5- monocytes provided an important factor required for HIV infection and rendered previously resistant cells sensitive to infection^29^. EGFRvIII was shown to be transferred from EGFRvIII-positive glioma cells to EGFRvIII-negative glioma cells via EVs. Interestingly, this transfer lead to the activation of EGFRvIII-mediated oncogenic pathways in the recipient cells^19^.

In addition to functional protein transfer, EV-mediated RNA delivery has also been implicated in pathophysiological processes. For example, the transfer of miRNA-19 from astrocytes to breast cancer cells via EVs has been shown to inhibit the expression of its target, PTEN. This reduction in PTEN expression is associated with a priming of the tumor microenvironment for metastatic outgrowth in vivo^30^. Furthermore, EV-mediated RNA transfer has also been shown to promote the induction of a metastatic phenotype in vivo. The transfer of EVs containing mRNA transcripts involved in metastasis and migration from highly metastatic MDA-MB-231 cells to less metastatic T47D cells induced a highly metastatic phenotype in cells that functionally took up MDA-MB-231-derived EVs^31^.

The fact that EVs have been shown to induce functional effects via the delivery of RNA and protein molecules provides great promise to the EV therapeutic field. To induce their functional influence, these EV-delivered molecules must reach their site of action within the cell in adequate quantities. Strikingly, the mechanisms responsible for EV-mediated delivery of these molecules have not yet been determined. Although some evidence that specific EV types are able to deliver their cargo via direct fusion with the plasma membrane is available^32–34^, EVs most likely deliver their cargo through a route similar to viruses via escape of endosomal compartments. It is anticipated that by shedding light on the EV cargo delivery process, the drug delivery field may be able to improve the delivery of therapeutic molecules to their intracellular sites of action.

## EV therapeutics

EVs can possess inherent tissue repair-promoting properties that may be exploited therapeutically. For example, researchers initially thought that the cardioprotective properties of mesenchymal stem cells (MSCs) resulted from their differentiation into healthy myocardium. However, the effects were later shown to be due to the paracrine effects of MSCs on the surrounding host tissues. Great interest in the therapeutic potential of EVs was generated when it was demonstrated that EVs are an important component of MSC-mediated cardioprotection^5^. Since this discovery, extensive preclinical research has revealed the utility of EVs as a therapeutic agent in liver^35^ and cardiac^36^ regenerative medicine.

EVs possess numerous advantages over cell-based therapies in the context of regenerative medicine. A major advantage is that EVs, depending on their source, may be less immunogenic than their parental cells, likely due to a lower abundance of transmembrane proteins such as MHC complexes on their surface^37^. Unlike live cells, EVs have a long shelf life and may be transported and stored for long periods. Furthermore, EVs do not replicate after injection. Thus, EVs present less risk of tumor generation and the transfer of latent viral pathogens.

EVs also possess numerous advantageous features as drug delivery vehicles that may help them to outperform synthetic drug carriers. Notably, EVs seem to possess an intrinsic ability to cross tissue and cellular barriers^38^. In addition, synthetic drug carriers, such as LNPs and polymeric micelles, suffer from high immunogenicity and toxicity^39^. As therapeutic EVs are derived from either autologous or benign biological sources, they are less likely to induce these adverse effects. In fact, MSC-derived EVs have been shown to exert inhibitory effects on immune responses^40^. Furthermore, some EVs may possess inherent targeting characteristics and display tropism for a particular cell or tissue^41^. This feature could be exploited to selectively deliver drugs to their intended targets while avoiding off-target effects.

EVs have been successfully utilized as a drug delivery system in preclinical settings. Recently, large quantities of MSC-derived EVs loaded with an anti-KRAS^G12D^ siRNA were produced by electroporation, which were capable of increasing survival in a mouse model of pancreatic cancer^42^. However, it should be noted that controversy exists regarding the effectiveness of electroporation for siRNA loading into EVs^43,44^. EVs have also been successfully used in preclinical regenerative medicine. For instance, MSC-derived EVs were capable of inducing osteochondral regeneration in a rat model of joint damage after an intra-articular injection^45^.

**Fig. 1:**
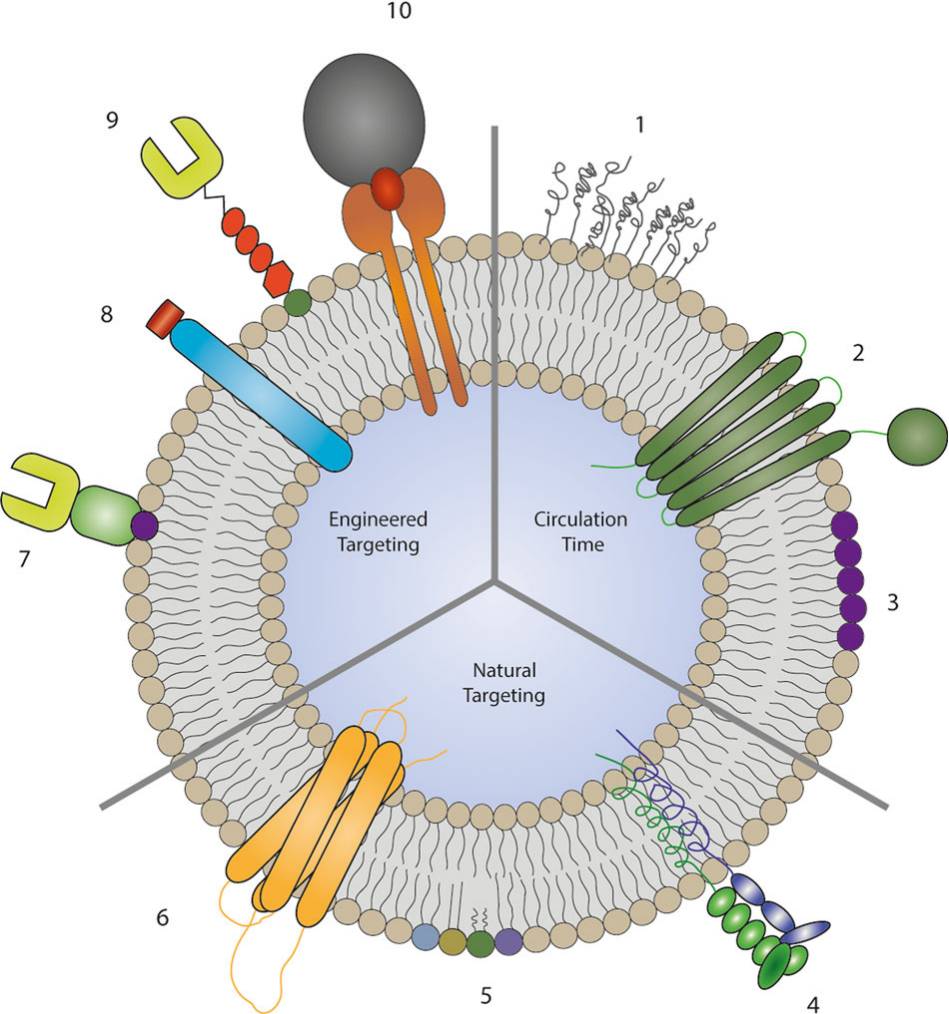
Naturally occurring or artificial features of EVs that alter the circulation time and targeting. The addition of polyethylene glycol (1) increases the circulation time^79^, the presence of CD47^47^ (2) inhibits uptake and clearance from the circulation by macrophages, while PS^58^ (3) is recognized by macrophages, leading to increased clearance. The integrin (4), lipid (5), and tetraspanin (6) compositions of EVs influence their natural targeting properties. These targeting properties are altered by the addition of targeting moieties anchored via the phosphatidylserine-binding C1C2 domains of lactadherin^82^ (7), the expression of lysosome-associated membrane protein 2 fusion proteins^38^ (8), glycosylphosphatidylinositol-anchored targeting moieties^81^ (9), and transferrin-conjugated magnetic particles bound to transferrin receptor expressed on EVs^93^ (10)

Numerous additional examples reveal the successful preclinical use of EVs, some of which are summarized later in this review. However, rather than focusing on these specific examples, a better understanding of their inherent general features, such as EV circulation kinetics, targeting, internalization, and intracellular trafficking routes, is needed to fully exploit these features of EVs for therapeutic purposes. In this review, we aim to summarize the current knowledge on these topics. We also aim to summarize methods for altering the characteristics of EVs to improve their therapeutic activity (Fig. 1).

## Circulation kinetics and biodistribution of EVs

The surface protein components of EVs are in part responsible for the determination of their circulation kinetics and biodistribution profile. This was clearly demonstrated by the observation that protease treatment of EVs prior to administration via intravenous injection resulted in a delayed clearance of EVs. Protease treatment also significantly reduced accumulation of EVs in the lungs, but did not alter uptake by macrophages^46^. However, according to other studies, EV surface proteins do indeed affect uptake by macrophages; for instance, CD47 has been found to inhibit EV uptake by macrophages^47^.

The biodistribution of therapeutic EVs is a crucial aspect of their efficacy and safety. In order to successfully make use of EVs for therapeutic delivery, an improved understanding of the biodistribution profile of exogenously administered EVs is pivotal. Using EVs labeled with a lipophilic dye, it was found that EVs accumulated primarily in the liver, spleen, and gastrointestinal tract of mice but that the biodistribution could be influenced by route of administration. Interestingly, the cellular origin of EVs also influenced this profile, suggesting that EVs from different cell sources possess different targeting characteristics. EVs from HEK293T cells also accumulated in subcutaneous tumors, a property which could be harnessed by EV-based anti-cancer therapeutics^48^. Another approach for labeling EVs for biodistribution studies is tagging EVs with luciferase. Using this method, it was found that luciferase-labeled EVs accumulated mostly in the lungs, liver, spleen and kidney^49^. Furthermore, *Gaussia princeps* luciferase-labeled EVs derived from a variety of mouse cell lines were used to demonstrate that intravenously administered EVs accumulated in the liver and were rapidly cleared from circulation in a partly macrophage-dependent manner^50^. Despite differences in biodistribution being observed between studies which have made use of EVs of different origins and different labeling techniques, a common consensus is that a large proportion of EVs are destined to accumulate in the liver and spleen, which could be exploited for specific therapeutic targeting of these organs.

In addition to the effects of the cell source, EVs with different sizes have also been shown to possess differing biodistribution profiles. Asymmetric flow-field fractionation has been used to isolate three subsets of EVs based on size. These subsets possessed differing molecular compositions and biophysical properties. Consistent with other observations, the majority of all subsets accumulated in the liver. However, the levels of accumulation in the bone and lymph nodes were significantly higher for large EVs as compared to the smaller subsets^51^. This is an interesting observation, but it remains unclear whether this is a result of EV size per se rather than a result of their differing molecular compositions.

None of these studies identified a significant distribution of EVs to the brain. However, there is some evidence that this may change during conditions of ischemic stroke. In a mouse model, strong uptake of cardiosphere-derived EVs was observed in the stroke penumbra. This phenomenon could be exploited for therapeutic targeting of tissues that are damaged after stroke^52^.

Despite the knowledge provided by these studies, an improved understanding of the factors that affect biodistribution would be highly beneficial for improving targeted EV delivery.

## Targeting and internalization—natural targeting properties of EVs

For an EV to exert its function, it must first bind to its recipient cell, and it is known that different EVs are capable of preferentially binding to specific target cell types. This inherent targeting ability of EVs is a feature that could be used to target EV drug delivery vehicles to their desired sites of action. Some of the features that are known to influence EV targeting are summarized in Table 1.

**Table 1:** An overview of natural EV features that influence targeting

EV Characteristic	Example	Reference
• Protein composition	Integrin profile	
	The display of different integrin complexes directs EVs to either the liver and brain or lungs.	^53^
	Tetraspanin profile	
	EVs containing the tetraspanin Tspan8 in complex with integrin α_4_ were shown to be selectively taken up by cells of the pancreas.	^54^
	CD63 + EVs were taken up by neurons and glial cells, while CD63- EVs bound only to the dendrites of neurons.	^55^
	Fibronectin	
	Fibronectin on MVEC-derived EVs mediates binding to OPCs via HPSGs.	^56^
• Lipid composition	PS-coated beads targeted and tethered to phagocytic cells via T-cell immunoglobulin mucin protein 4 receptor.	^57^
	PS-containing liposomes competitively inhibited EV uptake by murine macrophages.	^58^
• Glycan composition	Glycans on the EV surface directed EVs to CCR8-expressing GBM8 cells via a triple interaction with CCL18.	^59^
	Glycans enriched on MSC-derived EVs were involved in targeting EVs to HeLa cells via SIGLECs.	^60^
• Negative harge	Negatively charged PS- and phosphatidylglycerol-containing liposomes reduced EV uptake by murine macrophages, while neutral phosphatidylcholine liposomes did not.	^58^

As mentioned above, the protein contents of EVs alter targeting behaviors. For example, EVs displaying integrin α_6_ in complex with subunits β_1_ and β_4_ are directed to S100-A4-positive fibroblasts and surfactant protein C-positive epithelial cells in the lungs. EVs expressing subunits β_5_ and β_4_ are preferentially targeted to Kupffer cells in the liver and CD31-positive endothelial cells in the brain, respectively^53^.

The tetraspanin class of proteins is abundant on the surface of many EV types^18^, and their roles in EV targeting have been investigated. This class of proteins forms a diverse range of complexes with other tetraspanins and integrins, and these complexes have been shown to determine targeting behaviors. For instance, a complex of the tetraspanin Tspan8 with integrin α_4_ has been shown to selectively target EVs to cells in the pancreas^54^. In addition, CD63-positive EVs have been shown to target neuronal and glial cells, while CD63-negative EVs bound only to the dendrites of neurons^55^. Furthermore, fibronectin present on the EV surface has been implicated in targeting microvascular endothelial cell-derived EVs to oligodendrocyte precursor cells via an interaction with heparin sulfate proteoglycans^56^.

The EV lipid composition also influences targeting behavior, the clearest example being the targeting of macrophages via recognition of PS on the EV surface. The importance of PS in this interaction is confirmed by the observation that PS-coated beads are targeted and taken up by phagocytic cells via tethering by T-cell immunoglobulin mucin protein 4 receptor^57^. Furthermore, PS-containing liposomes have been shown to reduce EV uptake by competing with and thereby inhibiting their uptake by macrophages. This recognition of PS by macrophages is likely partially mediated by its negative charge, as the uptake of negatively charged phosphatidylglycerol-containing liposomes also inhibited uptake, while neutral phosphatidylcholine-containing liposomes did not^58^.

In addition to lipid and protein determinants of EV targeting, glycans on the EV surface also play a role in cell targeting or uptake. Glycans have been shown to direct EV targeting towards CCR8-positive glioblastoma cells via a triple interaction with the CCR8 ligand CCL18^59^, while glycans on MSC-derived EVs have been shown to direct EVs towards surface-bound sialic acid-binding immunoglobulins, such as lectin receptors, expressed on the surface of HeLa cells^60^.

## Targeting and internalization—mechanisms involved in the internalization of EVs

It is important to study the ways in which EVs are internalized as this may be crucial for functional outcomes as the route of EV internalization dictates the functional response or efficiency of cargo delivery.

There are multiple routes through which EVs can be internalized by recipient cells, including phagocytosis, macropinocytosis, and receptor-mediated endocytosis. These routes result in the formation of intracellular vesicles containing the internalized material, which may then be further processed or sorted for degradation^61^. EVs may also fuse directly with the cell membrane; however, few examples of this process have been reported^32^. Extensive research has been conducted into the routes of EV uptake, some of which is summarized in Table 2. Numerous uptake routes have been implicated in EV function, and the major route of uptake seems to depend on the cell type being studied.

**Table 2:** An overview of research into EV uptake routes

Cell types	Study method summary	Implicated uptake route(s)	Reference
PC12 cell donor to bone marrow-derived mesenchymal stromal cell recipient	K+depletion and siRNA-mediated knockdown to inhibit key proteins involved in specific uptake routes.	Clathrin-mediated endocytosis and macropinocytosis	^63^
U87 glioblastoma cell donor to human umbilical vein endothelial cell, mouse embryonic fibroblast and U87 cell recipients	Chemical inhibition of cholesterol synthesis to inhibit lipid raft formation.	Lipid raft-dependent endocytosis	^66^
Mutu -, Mutu I, and Mutu III donors to various epithelial cell line recipients	Chemical inhibition of endocytosis, caveolin knockdown and determination of the co-localization of labeled EVs with tagged components of endocytosis.	Clathrin-independent endocytosis	^64^
A431 cell donor to HeLa cell recipient	Chemical inhibition of cholesterol synthesis, tyrosine kinases, Na+/H+ exchange and phosphoinositide 3-kinase. The siRNA-mediated knockdown of various key proteins involved in specific endocytosis pathways was also employed.	Clathrin-independent endocytosis and macropinocytosis	^65^
HeLa cell donor to MIA PaCa-2, A431, and BxPC-3 cell recipients	Activation of macropinocytosis via stimulation of EGFR, CXCR4 and oncogenic Ras.	Macropinocytosis	^67^
Oli-neu cell donor to primary mouse oligodendrocyte, cortical neuron, astrocyte and microglial recipients	Chemical inhibition of macropinocytosis.	Macropinocytosis	^68^
DU145 cell donor to HeLa cell and primary lung fibroblast recipients	Chemical inhibitors of endocytosis and siRNA-mediated knockdown of key proteins involved in specific endocytosis pathways.	Macropinocytosis and fluid-phase endocytosis	^69^
H4 neuroglioma cell donor to H4 neuroglioma and Chinese hamster ovary cell recipients	Chemical inhibition of macropinocytosis and clathrin- and caveolin-mediated endocytosis	None	^95^
K562 and MT4 cell donors to Raw264.7 and NIH 3T3 cell recipients	Chemical inhibition of phagocytosis and siRNA-mediated knockdown of key proteins involved in phagocytosis.	Phagocytosis	^96^

Clathrin-mediated endocytosis is a well-understood route of uptake for extracellular material and involves the formation of clathrin-coated endocytic vesicles^62^. CME has been implicated in EV uptake, as its inhibition resulted in reduced uptake in bone marrow-derived mesenchymal stromal cells^63^. Clathrin-independent endocytosis, which involves the formation of caveolin-coated invaginations on the cell membrane^61^, is another route of EV uptake^64,65^. It should be noted that this caveolin-dependent endocytosis has also been identified as a negative regulator of EV uptake^66^. In addition, a major route of EV uptake is macropinocytosis. This involves the uptake of large quantities of extracellular fluid into a macropinosome in a process that depends on actin polymerization^62^. Numerous examples of EV uptake through this pathway have been reported^63,65,67–69^. Finally, phagocytic cells, such as macrophages, also take up EVs via phagocytosis^58^.

The uptake of lipoproteins is often associated with the existence of a specific receptor. For instance, the uptake of LDL is now well characterized, and a receptor has long since been identified^70^. Currently, researchers have not determined whether such a bona fide receptor exists for EVs in general, or at least for a particular EV subtype. All the aforementioned proteins are broadly involved in EV targeting and uptake but are neither sufficient nor absolutely required for EV uptake. The discovery of a putative uptake receptor for a specific type or subtype of EV would tremendously improve the design of engineered EV therapeutics.

The routes by which EVs are taken up are diverse and depend on the producer and recipient cell type. To successfully develop therapeutic EVs, it would be of great interest to determine which routes of uptake result in high levels of functional cargo delivery, so that therapeutic EVs could be steered towards this route.

## Intracellular trafficking of EVs

Following uptake, extracellular material is kept separate from the cytosol and enters the endosomal system in early endosomes (EEs). Most of the EE contents are destined for degradation in the acidic environment of the lysosomes, including internalized EVs^71^. However, for EVs to exert their function, their cargo must reach its intracellular site of action. The ability of EVs to at least partially avoid this degradative pathway was revealed by the observation that EVs follow a similar route to human immunodeficiency virus for dissemination after uptake in mature dendritic cells^72^. This observation is of great interest, as it indicates that EVs are capable of bypassing degradative uptake pathways, but this finding may be specific to dendritic cells. It should also be noted that protease treatment did not alter this post-uptake trafficking, suggesting that this observation may not be the result of EV-specific features. Despite these concerns, a mechanism for EV escape from degradative pathways after uptake likely exists, as it has been observed in numerous cases that EVs are capable of exerting functional effects via the delivery of their cargo.

To investigate possible routes of degradative escape, EVs labeled with CD63-GFP/CD63-mCherry were produced and followed after internalization by human primary fibroblasts, Huh7 and HEK293 cells. Post-uptake, EVs were seen to be surrounded by larger vesicles which were then trafficked towards ER filaments, where interactions with the ER were observed, as demonstrated by microscopy. A potential exchange of content at these interaction sites between the vesicles and the ER was proposed^73^. This hypothesis is strengthened by the fact that Rab5- and Rab7-positive endosomal vesicles are known to interact with the ER^74^. The mechanisms underlying this potential process of functional content release, which requires content crossing the exosomal and endosomal membranes, remain unknown. In regards to miRNAs and siRNAs, if EV- mediated delivery to the ER is possible, this could be a pathway for functionally altering gene expression.

An analysis of internalized PC12 cell-derived EVs labeled with lipophilic dyes and amino‐reactive fluorophores by live-cell fluorescence microscopy in PC12 cells revealed that EVs were first encapsulated in EEs, which then moved towards the perinuclear space, the location of endosomes as well as lysosomes. Here, it was observed that the labeled protein signal began to separate from the EV membrane signal within 3 h, indicating separation of transmembrane proteins and lipids in the EV membrane. Between 6 and 24 h, the signal of the lipid-labeled EVs was substantially reduced and not present in the perinuclear space, suggesting that the lipids were recycled and transported to other parts of the cell^75^. In contrast, the signal of the transmembrane proteins could still be observed in the perinuclear space, suggesting that much of the protein remained in the lysosomes. This implies that a large amount of membrane-bound EV cargo is degraded in the lysosomes post-uptake.

It should be noted that research into the post-uptake routes of EV trafficking is hindered by the recent finding that commonly used lipophilic dyes used for EV labeling, such as PKH26, form particles which are hard to distinguish from labeled EVs and colocalize with them in subcellular compartments^76^. In addition, EVs are often tracked with fluorescently labeled tetraspanin proteins^77^, which remain associated with membranes even when EV content delivery occurs. It seems evident that assessment of EV content release must involve the use of cytosolic EV markers and the development of new assays to study EV cargo transfer.

The evidence suggesting that EVs are able to escape endosomal degradative pathways post-uptake is of great interest, as this feature could be exploited for therapeutic delivery. However, the mechanisms by which EVs are capable of avoiding degradation are currently poorly understood. In order to fully utilize EVs as therapeutics it is necessary to determine the intracellular routes and mechanisms by which their cargo is delivered.

## Engineering approaches that impact circulation kinetics and biodistribution

Altogether, EVs clearly possess many potential advantageous features compared to synthetic delivery systems in terms of their intrinsic therapeutic properties and ability to deliver functional cargo. However, unmodified EVs suffer from rapid clearance and low accumulation in target tissues and cells^48^. Therefore, EVs have been modified in order to steer their delivery towards their target sites of action.

In order to increase circulation time and improve delivery to target tissues, EVs have been coated with polyethylene glycol (PEG) functionalised with anti-EGFR nanobodies. PEG is a hydrophilic polymer and is known to increase the circulation time of nanoparticles^78^. It was found that PEGylation increased EV circulation time and reduced nonspecific interactions with cells, while enhancing the nanobody-mediated interaction with EGFR-expressing cells^79^. Similarly, the effect of cloaking of the EV surface with streptavidin conjugated to PEG via its linkage to 1,2-bis(dimethylphosphino)ethane lipids has also been employed. The streptavidin component facilitated the conjugation of targeting components, which successfully altered EV biodistribution in mice^80^. It should be noted however, that the addition of PEG to the surface of liposomes is known to hinder their escape from the endosome after uptake, which may also apply to PEGylated EVs^78^.

Another approach used to increase circulation time is through the increased expression of CD47 on the EV surface. This protein has been shown to act in opposition to PS, which promotes the initiation of phagocytosis and subsequent removal from the circulation by macrophages^58^. The CD47 protein has been found on EVs from specific cell types and has been demonstrated to increase circulation time following intraperitoneal injection^47^. This feature could be exploited to prolong the circulation time of therapeutic EVs, and thus increase the window for targeted delivery to specific tissues.

## Engineering approaches that impact the targeting or internalization of EVs

A major challenge hindering the utilization of EVs in a therapeutic context is the difficulty in ensuring delivery to their sites of therapeutic action while avoiding accumulation at off-target sites. Nonspecific delivery decreases efficacy and may induce off-target effects.

The targeting properties of EVs can be influenced by genetic modification of producer cells. The first example of EVs targeted in such a way involved the fusion of lysosome-associated membrane protein 2 (Lamp2b) with the rabies viral glycoprotein peptide. Lamp2b is abundant on the surface of EVs, while the rabies viral glycoprotein peptide binds specifically to the acetylcholine receptor. It was found that this fusion protein conferred EVs with the ability to target neurons, oligodendrocytes and microglia within the brain after systemic injection^38^. EVs with engineered targeting abilities have also been produced by modification of producer cells to produce recombinant EGFR-specific nanobodies with glycosylphosphatidylinositol (GPI)-anchoring peptides. As EVs are enriched in GPI, nanobodies were highly enriched on the EV surface, which provided the EVs with targeting specificity for EGFR+cells^81^. While both of these methods successfully targeted EVs to their intended cells of action, the genetic modification of producer cells may be challenging to utilize for future production of therapeutic EVs, due to their time-consuming production and difficulties in applying them to cells derived from a patient’s own body fluids^41^.

The application of targeting components post-EV production is an attractive option, as targeting ligands can be applied in a controllable manner at high densities^41^. There are numerous recent examples of targeting capabilities being conferred by the addition of nanobodies and antibodies post-EV production. For example, the high concentration of PS in EV membranes has been exploited by fusing targeting proteins to the C1C2 domain of lactadherin, which binds to PS with high affinity. An anti-EGFR nanobody fused to C1C2 self-associated onto EV membranes and promoted EV uptake by EGFR+cells in a dose-dependent manner. Nontargeted control nanobody fusion proteins did not alter interactions with cells, and the addition of the fusion protein did not alter EV size or integrity, demonstrating this method’s suitability for therapeutic EV targeting^82^. A similar approach was exploited by fusing an anti-Her2 single-chain variable fragment to the C1C2 domain. This fusion protein was able to latch onto the surface of EVs and targeted the delivery of an mRNA encoding a prodrug converting enzyme to Her2+cells. Remarkably, when administered with the prodrug, these targeted EVs were capable of almost entirely halting the growth of orthotopic Her2+BT474 xenografts in vivo^83^.

In addition to antibodies and nanobodies, EVs have also been functionalized with targeting peptides postproduction. For example, a multifunctional peptide was able to anchor itself to the EV membrane has been used. This peptide also contained a sequence directed to the low density lipoprotein receptor which is expressed on the BBB and glioma cells and an apoptosis-inducing sequence. The peptide contained an ApoA-I mimetic sequence that associated with phospholipids and allowed incorporation onto the EVs via simple incubation. This approach allowed accumulation of systemically injected EVs in the brain and a successful targeting of methotrexate-loaded EVs to glioma cells in mouse models, resulting in an increase in survival in mouse models of glioma^84^.

Click-chemistry refers to a group of reactions which involve the conjugation of molecules in a modular fashion^85^. One such reaction is bio-orthogonal copper-free azide alkyne cyclo-addition, which was used to couple a cyclo Arg-Gly-Asp-D-Tyr-Lys peptide to the surface of MSC-derived EVs. This peptide binds with high affinity to integrin α_v_β_3_, which is expressed in ischemic reactive cerebral vascular endothelial cells. These engineered EVs targeted the ischemic regions of the brain in a murine artery occlusion model while EVs displaying a control peptide did not. In addition, the targeted EVs were able to reduce the inflammatory response via delivery of their loaded curcumin cargo. The approach used in these experiments was robust, rapid, and scalable and could be applied to many other targeting peptides^86^. In addition to the two described here, there are numerous further examples of targeting peptides being linked to the EV surface, which are described in Table 3.

**Table 3:** Examples of engineering EV targeting by the addition of peptides

Targeting peptide	Linkage method	Result	Reference
RGERPPR— a specific peptide for the neuropilin-1 receptor expressed specifically on glioma and tumor vascular endothelial cells.	Click Chemistry (cyclo-addition reaction of sulfonyl azide).	EVs could cross the BBB and target glioma cells to deliver therapeutic payload, resulting in increased survival in a murine model of glioma.	^97^
M12—a muscle targeting peptide	Using phage display, the CP05 peptide, which binds to the extracellular loop of CD63 with high affinity, was identified. Fusion of targeting peptides to CP05 allowed coating of the EV surface via CP05-CD63 interaction.	Targeted EVs successfully delivered splice-correcting oligomers to muscle in a dystrophin-deficient mouse model of muscular dystrophy.	^98^
RGD—specifically binds to integrin α_v_β_3_ expressed on the surface of angiogenic blood vessels.	RGD anchored to EV surface via linkage to PEG-lipid which self-assembles into EV membrane.	EVs were targeted to α_v_β_3_ cells in zebrafish and promoted angiogenesis.	^99^
CTP—cardiac targeting peptide	Recombinant CTP-Lamp2b expressed in donor cells.	15% increase in delivery to mouse hearts after intravenous injection.	^100^

In addition to their use as mediators of cell targeting, peptides have also been used in order to promote the uptake of EVs into cells. For example, Nakase et al. decorated the surface of EVs with an arginine-rich micropinocytosis-inducing peptide via a sulfo-N-ε-maleimidocaproyl-oxysulfosuccinimide ester linkage. This modification substantially increased uptake into CHO-K1 cells. Interestingly, this increase in uptake was associated with an improved delivery of the loaded ribosome-inactivating cytotoxic saporin protein^77^. The application of this cell-penetrating peptide may be a suitable method for improving the intracellular delivery of therapeutic EV cargo. Furthermore, the same group were also able to promote EV uptake by inducing the clustering and activation of EGFR. Activation of this receptor is known to induce EV uptake via the promotion of macropinocytosis^67^. Recipient HeLa cells were engineered to express a modified form of EGFR which bound with high affinity to a stearylated peptide which could be anchored to the EV membrane with high affinity. These modified EVs were capable of inducing receptor clustering and promoting uptake via macropinocytosis and an associated increase in the activity of loaded saporin^87^.

In addition to the use of targeting peptides, nanobodies, and antibodies, pseudotyping has also been used to promote EV uptake. Pseudotyping is a well-studied method commonly used in virology to alter the tropism of a viruses via the introduction of foreign protein tropism determinants from another distinct viral species^88^. Meyer et al. utilized this method by expressing vesicular stomatitis virus glycoprotein in HEK293 cells. This protein was chosen because it is often used to increase the cell tropism and transduction efficiency of therapeutic retroviral vectors. The vesicular stomatitis virus glycoprotein was expressed in the membranes of EVs, and its ectodomain was responsible for inducing a large increase in uptake in several different cell types^89^.

Most of the research aiming to alter EV tropism has relied on the addition of proteins or peptides. However, augmented targeting of specific liver cells and an increase in EV uptake has been achieved by modifying the surface of EVs with cationized pullulan. This polysaccharide is known to bind an asialoglycoprotein receptor expressed specifically on hepatocytes. The modification of the surface of MSC-derived EVs with this molecule promoted an increase in uptake in vitro. Furthermore, in a rat model of liver damage, pullulan-modified EVs were targeted to the liver and significantly improved clinical parameters of liver function^90^.

Another innovative approach to direct EV targeting, which does not rely on proteins or peptides, is through the addition of nucleic acid aptamers which are able to bind target molecules. This approach was recently utilized to direct HEK293T-derived EVs towards prostate cancer cells. In this study, the three-way-join (3WJ) of the bacteriophage phi29 motor packaging RNA was used as a building block to which cholesterol and a targeting aptamer specific for prostate-specific membrane antigen (PSMA) were fused. When cholesterol was bound to the “arrowhead”, the 3WJ was loaded inside of the EVs; however, when cholesterol bound to the “arrowtail”, the 3WJ was displayed on the EV surface. This observation was exploited to produce EVs which contained a therapeutic anti-survivin siRNA and displayed a PSMA-binding RNA aptamer on their surface. The addition of this aptamer increased uptake in PSMA+cell lines and reduced tumor growth in mouse xenograft models^91^. In addition to RNA aptamers, DNA aptamers have also been used to direct EV targeting. The DNA aptamer AS1411 specific for nucleolin, was used to target the delivery of therapeutic siRNAs to cells positive for this protein expressed on the surface of breast cancer cells. The modification of EVs with this aptamer promoted the delivery to tumors in vivo, which was associated with an inhibition of tumor growth^92^.

EVs have also been targeted without the addition of targeting ligands, but instead by the addition of magnetic particles. In this way, the biodistribution of EVs decorated with magnetic particles can be controlled using directed magnetic fields. For example, the abundance of transferrin receptor on blood-derived EVs was exploited to coat their surface with transferrin-conjugated superparamagnetic nanoparticles. Post intravenous administration, the biodistribution of these particles was successfully controlled and targeted towards murine tumors using external magnets^93^.

Although many examples of the targeting of EVs to specific cell types or tissues exist, relatively few examples of EVs being targeted towards specific uptake routes or subcellular locations have been reported. EVs have been shown to be directed towards specific uptake routes when decorated with anti-Her2 antibodies. Anti-Her2 directed EVs displayed different co-localization patterns to wild-type EVs and EVs coated with a nonspecific antibody. Wild-type EVs mainly co-localized with a protein known to be taken up via caveolae-mediated endocytosis, while nonspecific antibody-coated EVs co-localized with most strongly with dextran, which is taken up by macropinocytosis. When anti-Her2-targeted EVs were analyzed, they co-localized with markers known to be taken up by macropinocytosis, caveolin-mediated, and clathrin-mediated endocytosis^94^.

Altogether, these observations provide evidence that it is possible to tune EV routes of uptake and subsequent subcellular destination. This tuning may substantially increase the efficiency of RNA therapeutic delivery by avoiding degradative uptake routes.

## Conclusions and perspectives

In recent years, the importance of EVs as mediators of intercellular communication has been reported, and it has been demonstrated that EVs possess several features which make them amenable to therapeutic use. For these reasons, the EV field is undergoing a period of rapid growth. This growth has been associated with discoveries which have elucidated some of the targeting abilities, uptake routes, and biodistribution profiles of EVs.

Despite these recent advances, many aspects of their fundamental biology remain to be elucidated. In order to effectively utilize EVs in a therapeutic context, it would be highly advantageous to first gain an improved understanding of aspects of their biology.

Although there have been many successful attempts to alter the biodistribution and cell-targeting properties of EVs, there has been relatively little work undertaken with the purpose of increasing the delivery of EV cargo to its intracellular site of action. It is possible that a large proportion of EVs taken up by a cell are destined for degradation. Therefore, the identification of features which would allow for improved cargo escape would be highly advantageous.

In addition, the routes of EV uptake are highly diverse and vary according to cell and EV types. It is possible that a particular uptake route may result in the delivery of a greater amount functional cargo to the recipient cell than other routes. If EVs were steered towards cellular uptake mechanisms that result in the increased functional delivery of cargo, the efficacy and efficiency of EV-mediated therapeutic strategies would be substantially improved.

It is also known that EV preparations contain a range of EV subtypes, which vary in terms of subcellular site of origin, size, and protein markers. At present it is extremely challenging to separate these subtypes for functional analysis, and further characterization of the physical and functional properties of subpopulations found in the heterogeneous population of EVs is still ongoing. However, it is possible that the research of certain subtypes for therapeutic purposes could result in the discovery of additional advantageous modifications or strategies for EV-based therapeutics.

In conclusion, many aspects of the uptake, biodistribution, targeting, and trafficking of EVs have been elucidated. A large amount of successful research has also been undertaken into methods by which these features may be altered to produce effective therapeutic EVs. However, in order to translate these findings into clinically successful therapeutics, further research into the underlying biology of EV-mediated cargo transfer and processing is required.
